# Guiqi Baizhu prescription attenuates 5-FU-induced intestinal mucositis by targeting IKKβ to inhibit M1 macrophage polarization

**DOI:** 10.1186/s13020-026-01406-z

**Published:** 2026-07-16

**Authors:** Yu-Cen Zhou, Ya-Ling Li, Jing Ma, Junjie Li, Qi Si, Shunzhi Wang, Dan Huang, Xiao-Xia Wa, Hui Cai, Yong-Qi Liu

**Affiliations:** 1 NHC Key Laboratory of Diagnosis and Therapy of Gastrointestinal Tumor, Gansu Provincial Hospital, Lanzhou, Gansu 730000 People’s Republic of China; 2 Key Laboratory of Dun Huang Medical and Transformation, Ministry of Education of The People’s Republic of China, Gansu University of Chinese Medicine, Lanzhou, Gansu 730000 People’s Republic of China; 3 Gansu University Key Laboratory for Molecular Medicine & Chinese Medicine Prevention and Treatment of Major Diseases, Gansu University of Chinese Medicine, Lanzhou, Gansu 730000 People’s Republic of China

**Keywords:** Guiqi Baizhu prescription, Chemotherapy-induced intestinal mucositis, Macrophages polarization, IKKβ, NF-κB

## Abstract

**Background:**

Chemotherapy-induced intestinal mucositis (CIM), particularly that induced by agents such as 5-fluorouracil (5-FU), frequently leads to chemotherapy discontinuation. However, effective treatment options remain limited. Guiqi Baizhu prescription (GQBZP), a traditional Chinese medicine formula, has been reported to possess anticancer, analgesic, and anti-inflammatory activities.

**Purpose:**

This study aimed to evaluate the therapeutic efficacy of GQBZP against 5-FU-induced intestinal mucositis (IM) and to clarify its underlying molecular mechanisms and material basis.

**Methods:**

The protective effects and mechanistic actions of GQBZP were investigated using a murine model of 5-FU-induced IM. Potential IKKβ-targeting compounds within GQBZP were screened through virtual docking combined with CCK-8 assays and subsequently evaluated in 5-FU-stimulated human intestinal epithelial cells (HIECs) and lipopolysaccharide/interferon-γ (LPS/IFN-γ)-stimulated THP-1 macrophages. Target specificity and binding characteristics were further validated by molecular dynamics (MD) simulations, surface plasmon resonance (SPR) analysis, and experiments using IKKβ-overexpressing HEK293T cells.

**Results:**

In vivo experiments demonstrated that GQBZP significantly alleviated 5-FU-induced IM by suppressing M1 macrophage polarization within intestinal tissues and restoring intestinal barrier integrity, effects closely associated with modulation of the IKKβ/NF-κB signaling pathway. Virtual screening and CCK-8 assays identified five IKKβ-targeting compounds in GQBZP: Rhamnocitrin, Toralactone, Naringenin, Liquiritigenin, and Carvacrol. These compounds markedly reduced apoptosis and pro-inflammatory cytokine production in 5-FU-treated HIECs. In addition, they inhibited M1 macrophage polarization and cytokine release in LPS/IFN-γ-stimulated THP-1 cells, accompanied by attenuation of IKKβ/NF-κB pathway activation. MD simulations, SPR assays, and functional studies in IKKβ-overexpressing HEK293T cells further confirmed that Toralactone directly binds to and suppresses IKKβ activation, whereas Rhamnocitrin modulates IKKβ activity through an indirect regulatory mechanism.

**Conclusion:**

GQBZP alleviates 5-FU-induced IM by promoting recovery of the intestinal epithelial barrier and inhibiting M1 macrophage polarization through an IKKβ/NF-κB-dependent mechanism, thereby exerting synergistic anti-inflammatory effects. Toralactone was identified as a key active constituent responsible for direct inhibition of IKKβ-mediated M1 polarization. These findings suggest that targeting IKKβ to regulate macrophage polarization represents a promising therapeutic strategy for the management of CIM.

**Supplementary Information:**

The online version contains supplementary material available at 10.1186/s13020-026-01406-z.

## Introduction

Chemotherapy, a primary treatment for cancer, frequently induces intestinal mucositis, which often necessitates the discontinuation of chemotherapy and significantly diminishes patients’ quality of life [1]. The chemotherapeutic agents most commonly linked to severe IM and diarrhea include 5-FU, Capecitabine (a 5-FU prodrug), Irinotecan (CPT-11), Methotrexate, and Tegafur [2]. Current clinical guidelines for managing CIM primarily recommend lipid-based compounds and vitamins as nutritional interventions; however, their therapeutic efficacy remains limited [3]. This study aims to investigate a novel treatment strategy specifically targeting 5-FU-induced IM.

Chemotherapy drugs, such as 5-FU, induce injury to intestinal mucosal cells primarily through DNA damage, oxidative stress, amplified inflammatory signaling cascades, and dysregulation of the immune microenvironment. The activation of the NF-κB signaling pathway plays a central role in this process, causing inflammatory injury to intestinal epithelial cells and triggering intestinal immune dysregulation characterized by macrophage infiltration and aberrant polarization [4]. Within inflamed intestinal tissue, M1 macrophages secrete pro-inflammatory mediators including IL-1β, IL-6, TNF-α, and iNOS [5–9], thereby exacerbating epithelial injury. Recent studies have demonstrated that the NF-κB signaling pathway is closely involved in regulating the integrity of intestinal tight junctions and cytokine expression in 5-FU-induced IM [10, 11]. IKKβ activates the canonical NF-κB pathway by promoting nuclear translocation of the p50/p65 heterodimer and initiating transcription of pro-inflammatory factors such as interferons and interleukins [12]. Importantly, the enzymatic activity of IKKβ makes it a highly attractive and tractable therapeutic target. As a kinase, it is susceptible to modulation by small-molecule inhibitors. Selective inhibition of IKKβ effectively blocks canonical NF-κB activation downstream of multiple stimuli, including tumor necrosis factor-alpha (TNF-α), IL-1, and TLR ligands, offering a powerful strategy to attenuate pathological NF-κB-driven processes.

GQBZP, derived from the Astragali Decoction documented in Zhu Su’s Puji Fang (Universal Relief Manual) during the Ming Dynasty, is composed of Astragalus membranaceus, Atractylodes macrocephala, Angelica sinensis, Paeonia lactiflora, Citrus reticulata peel, Rheum palmatum, and Glycyrrhiza uralensis. This formulation is traditionally recognized for its effects in fortifying the spleen and removing blood stasis, and has demonstrated clinical benefits in alleviating diarrhea in cancer patients following chemotherapy. Previous studies from our group have shown that GQBZP provides significant protective effects against radiation therapy-induced intestinal edema and damage to the intestinal barrier [13]. Additionally, GQBZP markedly attenuates Ara-C-induced ileal injury by suppressing M1 macrophage polarization [14]. It is noteworthy that although both 5-FU and Ara-C are antimetabolites that inhibit DNA synthesis, they are used to treat distinctly different tumor types. Moreover, while both drugs can cause gastrointestinal side effects, Lu et al. [15] reported that the jejunum is the most severely affected site in 5-FU-induced intestinal injury. In contrast, our previous studies indicate that Ara-C predominantly damages the ileum [14].

Therefore, this study aimed to investigate the regulatory effects of GQBZP on the intestinal mucosal barrier and macrophage polarization, with the goal of elucidating its underlying mechanisms of action and identifying its key active components. These findings will provide new insights into the potential use of GQBZP for the treatment of CIM.

## Materials and methods

### Animals and materials

All experimental protocols were approved by the Animal Experimental Ethics Committee of Gansu University of Traditional Chinese Medical Practice (Approval No. 2022–159). Male C57BL/6 mice (20–25 g, 6–8 weeks old, SPF) [Certificate No. SYXK (gan) 2020–0009] were obtained from SPF (Beijing) Biotechnology Co., Ltd. The production license for this product was issued in Beijing, China, on January 10, 2019 (License No. SCXK: 2019–0010). The animals were housed at 22 ± 3 °C under a 12-h light/dark cycle, with free access to food and water. Body weight and food intake were recorded daily throughout the study.

5-FU (S1209) and LY2409881 (S7697) were purchased from Selleck Chemicals LLC (Houston, USA). The GQBZP solution used in animal experiments was provided by the Affiliated Hospital of Gansu University of Traditional Chinese Medicine (TCM, Lanzhou, China). Lipopolysaccharide (LPS, L2630) was obtained from Sigma-Aldrich (St. Louis, MO, USA), and interferon-gamma (IFN-γ, 300–02-20UG) was sourced from Peprotech (Rocky Hill, NJ, USA). Rhamnocitrin (HY-N1353), Toralactone (HY-N7617), Carvarol (HY-N0711R), Scopoletin (HY-N0342), and TNF-α (HY-P1875) were procured from MedChem Express (Monmouth Junction, NJ, USA). Akridin (CAS 260–94–6), Naringenin (CAS 480–41–1), and Liquiritigenin (CAS 578–86–9) were obtained from Meilunbio (Dalian, China). Phorbol 12-myristate 13-acetate (PMA, P6741) was purchased from Beijing Solarbio Science & Technology Co., Ltd. (Beijing, China). Recombinant human IKKβ protein was sourced from Proteintech Group, Inc. (Wuhan, China). Antibodies used in this study included: p-IKKβ (ab194519), F4/80 (GR3327073-5), iNOS (ab178945), and goat anti-rat secondary antibody iFluor^™^ 647 (GR3286150-2) from Abcam (Cambridge, MA, USA); IKKβ (GTX105690), Occludin (821,604,845), and ZO-1 (43,362) from GeneTex (San Antonio, TX, USA); NF-κB p65 (ET1603-12), phospho-NF-κB p65 (HA723223), IL-1β (ET1701-39), IL-6 (R1412-2), Ki-67 (SR00-02), and GAPDH (ET1601-4) from Huabio (Hangzhou, China); F4/80 (123,107) and CD163 (155,305, 333,610) from BioLegend (San Diego, CA, USA); and CD86 (F1108602, F2108602) and CD11b (F11011B04) from MultiSciences Biotech (Hangzhou, China).

### Animal experiment

**Fig. 1:**
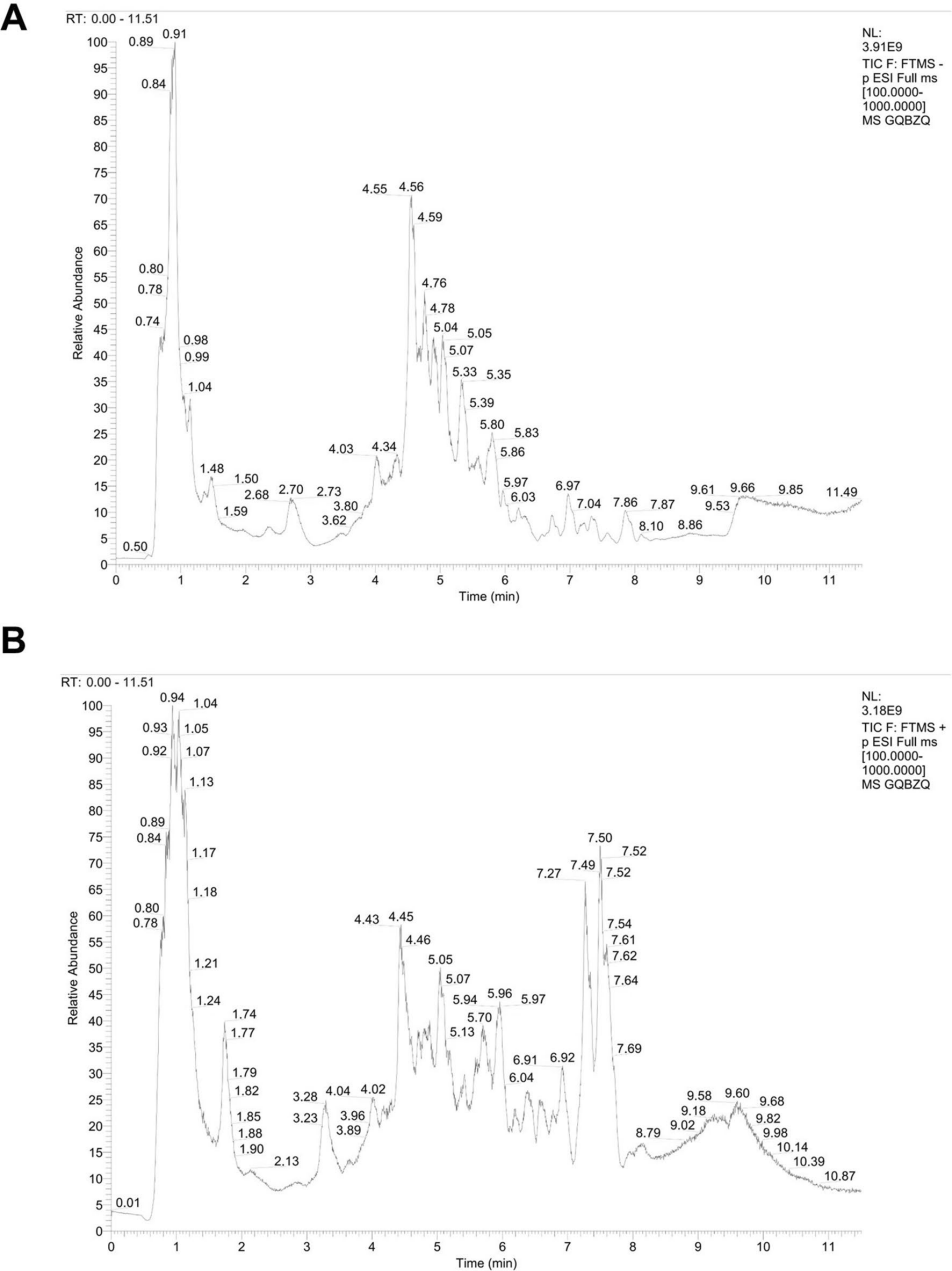
Tentative identification of chemical constituents in the water decoction of GQBZP by UPLC-MS/MS. **A** Total ion chromatogram obtained in the positive ion mode. **B** Total ion chromatogram obtained in the negative ion mode

The clinical adult dose of GQBZP (69 g crude herbs) was converted to 8.97 g/kg in mice (equivalent to 0.45 g/mL decoction) using a human‑to‑mouse dose conversion factor of 9.1, which was set as the medium dose. The low dose (4.45 g/kg, 0.22 g/mL) and high dose (17.94 g/kg, 0.90 g/mL) were also used. The chemical composition of the aqueous GQBZP decoction was analyzed by UPLC-MS/MS, and total ion current (TIC) chromatograms were obtained in both positive and negative ionization modes, as shown in Fig. 1. This analytical method allowed for the identification of multiple representative components of GQBZP. A detailed summary of the identified compounds is provided in Table S1.

Following a 7-day acclimatization period, mice were randomly assigned to five experimental groups (n = 9 per group): Control (saline, intraperitoneal), 5-FU (50 mg/kg, intraperitoneal), and three 5-FU + GQBZP groups (50 mg/kg 5-FU, intraperitoneal) combined with low-dose GQBZP (4.45 g/kg, intragastric), medium-dose GQBZP (8.97 g/kg, intragastric), and high-dose GQBZP (17.94 g/kg, intragastric). The 5-FU dose used to induce IM was selected based on multiple published studies [16–19], ensuring the stability and reproducibility of the model. To establish the mucositis model, mice in the 5-FU and 5-FU + GQBZP groups received daily intraperitoneal injections of 5-FU (50 mg/kg) for 7 consecutive days. Concurrently, the three 5-FU + GQBZP groups received daily intragastric gavage of corresponding doses of GQBZP decoction from day 1 through day 7. Body weight and diarrhea scores were recorded daily. After the final treatment, mice were fasted for 24 h, anesthetized, euthanized, and intestinal tissues were collected for subsequent analyses.

### Cell culture

Human small intestinal epithelial cells (HIEC) were kindly provided by the Stem Cell Bank of the Chinese Academy of Sciences (Shanghai, China). Human embryonic kidney 293T (HEK293T) cells were donated by the Key Laboratory of Molecular Medicine of Major Diseases and Prevention and Treatment with TCM of Gansu Province. Both cell lines were cultured in Dulbecco’s modified eagle medium (VivaCell) supplemented with 10% fetal bovine serum (FBS; VivaCell) at 37 °C in a humidified atmosphere containing 5% CO_2_. Human monocytic leukemia THP-1 cells and complete culture medium were obtained from Shanghai Fuheng Biotechnology Co., Ltd. (Shanghai, China). THP-1 cells were cultured in RPMI-1640 medium supplemented with 0.05 mM β-mercaptoethanol, 10% FBS, and 1% penicillin–streptomycin at 37 °C with 5% CO_2_. To induce differentiation, THP-1 cells were treated with 100 ng/mL PMA for 48 h to generate M0 macrophages. Subsequently, M0 macrophages were stimulated with 100 ng/mL LPS and 20 ng/mL IFN-γ for 48 h in PMA-containing medium to induce M1 polarization [20]. This model effectively activates the NF-κB signaling pathway and is suitable for mechanistic studies of therapeutic compounds [21].

### Immunohistochemistry assay

Following deparaffinization and rehydration, jejunal tissue sections underwent antigen retrieval using citrate buffer. Endogenous peroxidase activity was blocked with 3% hydrogen peroxide, followed by a 1-h incubation with goat serum to prevent nonspecific binding. The sections were then incubated overnight at 4 °C with primary antibodies against IL-1β (1:500), IL-6 (1:500), and Ki-67 (1:2000). After washing, secondary antibodies were applied and incubated at room temperature for 10 min, followed by a 10-min incubation with streptavidin–peroxidase conjugate. Immunoreactivity was visualized using DAB chromogen, and nuclei were counterstained with hematoxylin. Stained sections were examined and imaged using a light microscope.

### Immunofluorescence assay

Cells and jejunal tissue sections were fixed in 4% paraformaldehyde for 20 min, then permeabilized and blocked with PBS containing 0.3% Triton X-100 and 10% goat serum for 1 h. Samples were subsequently incubated overnight at 4 °C with the following primary antibodies: ZO-1 (1:1000), Occludin (1:200), F4/80 (1:1000), iNOS (1:500), p-IKKβ (1:200), IKKβ (1:500), phospho-p65 (p-p65, 1:200), and p65 (1:500). Multi-label staining was performed following the protocol provided with the TSA multi-color fluorescence labeling kit (NEFP4100A, Histovabio, Beijing, China). For quantitative analysis, images from three mice per group or five biological replicates per cell experiment were evaluated.

### Flow cytometry

Mouse jejunum was isolated to prepare single-cell suspensions. After removing the mesenteric adipose tissue, jejunal segments were incubated in PBS at 37 °C for 20 min to facilitate detachment of epithelial cells. The resulting cell suspension was sequentially filtered, centrifuged, and enzymatically digested using a solution containing collagenase (11088858001, Roche, Basel, Switzerland) + Dispase II (42513–33–2, Coolaber, Beijing, China). Following resuspension and centrifugation, cells were separated by density gradient centrifugation using Percoll medium (MeilunBio, China). Approximately 1 × 10^5^ cells from the intermediate layer were collected for subsequent analyses.

After pharmacological treatment, adherent cells were harvested by trypsinization and washed three times with PBS. Apoptosis rates were assessed using an Annexin V-APC/7-AAD detection kit (AP105, Multi Sciences, Hangzhou, China). Polarization of LPS/IFN-γ-treated THP-1 cells was evaluated by staining with CD86-PE (Multi Sciences) and CD163-APC (BioLegend, USA). All samples were analyzed by multicolor flow cytometry using a BD LSRII system (BD Biosciences, USA), following the manufacturers’ standardized protocols.

### ELISA

Conditioned cultures were centrifuged at 2–8 °C and 2000–3000 rpm for 20 min. Supernatants were collected and stored at −80 °C. Protein levels of TNF-α, IL-1β, and IL-6 were quantified using commercial ELISA kits (EK182, EK101B, EK106; Multi Sciences, Hangzhou, China) according to the manufacturers’ protocols. Each experimental group included five independent biological replicates.

### RT-qPCR

Fresh jejunal tissues (30 mg) were homogenized in 1 mL of ice-cold lysis buffer (LB) using a cryogenic tissue homogenizer at high speed. Following centrifugation at 12,000 rpm for 7 min at 4 °C, total RNA was extracted from the supernatant and reverse-transcribed into cDNA using the reverse transcription kit (AG11705, Accurate Biology, Hunan, China). Quantitative real-time PCR was conducted to analyze the mRNA expression of target genes IL-1β and IL-6, with GAPDH serving as the reference gene. Primers for IL-6 and IL-1β were designed and synthesized by Beijing Ruiboxing Biotechnology Co., Ltd. and purified via ULTRAPAGE (Table S2).

### Assay of serum D-lactate

Mouse serum samples and D-lactate calibration standards (50 μL each) were loaded in duplicate into a designated 96-well plate. After adding 50 μL of chromogenic working solution (13,811, AAT Bioquest, Sunnyvale, CA, USA), the plate was incubated at 25 °C for 30–120 min in the dark. Absorbance was measured at 575 nm (primary) and 605 nm (reference) using a microplate reader. D-lactate concentrations (μM) were calculated by interpolation from a four-parameter logistic standard curve.

### Western blotting

Cell samples were prepared using standard protocols and separated by SDS-PAGE. Proteins were then transferred onto PVDF membranes using Tris–glycine buffer and probed with specific primary antibodies. Blots were visualized using the ChemiDoc system (BIO-RAD), and protein band intensities were quantified with ImageJ software. The primary antibodies used included p-IKKβ (1:1000), IKKβ (1:1000), p-p65 (1:500), p65 (1:1000), and GAPDH (1:2000), followed by incubation with corresponding secondary antibodies at a 1:5000 dilution.

### Molecular docking and protein–ligand interaction profiler (PLIP)

The IKKβ protein structure (PDB ID: 4KIK) was retrieved from the RCSB Protein Data Bank and prepared in PDBQT format to serve as the receptor for docking studies. Chemical constituents of GQBZP were obtained from the HERB database (http://herb.ac.cn/). Structural preparation of the small-molecule compounds from GQBZP was performed using the LigPrep module in Schrödinger 2019. Low-energy conformations were generated using the MMFFs force field, and ionization states were assigned at pH 7.0 ± 2.0 with Epik 2.8 to ready the ligands for docking. Drug-likeness of the compounds was evaluated according to Lipinski’s rule of five.

Ligand structures, including Carvacrol, Kaempferol, Liquiritigenin, Naringenin, Rhamnocitrin, and Toralactone, were downloaded from PubChem and converted from MOL2 to PDBQT format using OpenBabel v2.4.1 [22]. Hydrogen atoms were added, partial charges assigned, and energy minimization performed using AutoDock Tools. The co-crystallized ligand K-252A in IKKβ was used to define the active site. The docking grid box, with dimensions 50 × 50 × 50 Å, was centered at coordinates X: 43.607, Y: 9.184, Z: −64.344 using AutoDock Tools. Protein–ligand interactions were analyzed with the PLIP and visualized using PyMOL v2.6 [23].

### Molecular dynamic (MD) simulation

MD simulations of the protein–ligand complexes obtained from molecular docking were performed for 100 ns using GROMACS v2022.03 [24] with the CHARMM36 force field. Initial structures were prepared by converting the protein–ligand complexes from PDB to GRO format. Ligand parameterization was conducted using AmberTools22, employing the generalized amber force field (GAFF) [25]. Charge calculations were performed using Gaussian 16W. Long-range electrostatics were treated with the particle mesh Ewald method, and bond constraints were applied via the LINCS algorithm.

Subsequent analyses included calculations of root-mean-square deviation (RMSD), root-mean-square fluctuation (RMSF), radius of gyration (Rg), solvent-accessible surface area (SASA), and hydrogen bond (H-bond) formation. Based on RMSD and Rg profiles, conformational free energy analysis was conducted using built-in GROMACS tools (“g_sham” and “xpm2txt.py” scripts). Binding free energies were calculated using the molecular mechanics/Poisson-Boltzmann surface area (MM/PBSA) method implemented in "MMPBSA.py v16.0" [26].

### Surface plasmon resonance (SPR)

SPR measurements were performed using a Biacore 8 K instrument (GE Healthcare). Purified IKKβ protein (20 μg/mL in 10 mM sodium acetate buffer, pH 4.5) was immobilized on a Series S CM5 sensor chip via amine coupling, achieving an immobilization level of approximately 12,600 response units (RU). PBS containing 1% DMSO (pH 7.4) was used as the running buffer. All experiments were performed in one independent experimental run.

For binding analysis, Rhamnocitrin (eight concentrations ranging from 7 to 500 μM) and Toralactone (eight concentrations ranging from 0.16 to 10 μM) were prepared in running buffer and injected simultaneously at a flow rate of 20 μL/min. Association and dissociation phases were monitored for 100 s and 180 s, respectively, at 25 °C. Double referencing was applied to all sensorgrams by subtracting blank buffer injections and signals from a reference flow cell to correct for nonspecific binding and bulk refractive index changes.

Sensorgram data were globally fitted to a 1:1 Langmuir binding model using Biacore 8 K Evaluation Software (GE Healthcare) to determine the association rate constant (ka), dissociation rate constant (kd), and equilibrium dissociation constant (KD = kd/ka). The quality of the fit was validated by a χ^2^ value below 10 RU^2^.

### Method for IKKβ overexpression in HEK293T cells

The overexpression plasmid pLV3-CMV-IKBKB (human)−3 × FLAG-mCherry-Puro was diluted in ultrapure water. Solution A was prepared by mixing 100 μL of serum-free basal medium with 2 μg of plasmid DNA, followed by thorough pipetting. Solution B was prepared by mixing 100 μL of serum-free basal medium with 6–8 μL of PEI 40 K Transfection Reagent (G1802-1ML, Servicebio, Wuhan, China) and mixed thoroughly by pipetting. Solution B was then added to Solution A, gently mixed, and incubated at room temperature for 15 min to allow formation of DNA-PEI complexes. The transfection complexes were added to a 6-well plate containing pre-seeded cells in 1.5 mL of serum-free medium. The plate was gently rocked back and forth, as well as sideways, to ensure even distribution of the transfection reagent. Cells were incubated at 37 °C in a humidified CO₂ incubator. After 4 h, the medium was replaced with complete growth medium. Cells were cultured in the presence of puromycin for 48 h, after which the medium was refreshed before proceeding with further experimental validation. The pLV3-CMV-IKBKB (human)−3 × FLAG-mCherry-Puro plasmid was synthesized by Lanzhou Miaoling Biotechnology Co. The corresponding gene sequence is available in the NCBI database under accession number NM_001556.3.

### UPLC-MS/MS

Chromatographic separation was performed using a Thermo Vanquish ultra-high performance liquid chromatography system (Thermo Fisher Scientific, USA) equipped with an ACQUITY UPLC^®^ HSS T3 column (2.1 × 100 mm, 1.8 µm; Waters, Milford, MA, USA). The flow rate was set to 0.3 mL/min, the column temperature maintained at 40 °C, and the injection volume was 2 μL. For positive ion mode, the mobile phase consisted of 0.1% formic acid in water (A2) and 0.1% formic acid in acetonitrile (B2), with the following gradient elution: 0–1 min, 8% B2; 1–8 min, 8–98% B2; 8–10 min, 98% B2; 10–10.1 min, 98–8% B2; and 10.1–12 min, 8% B2. For negative ion mode, the mobile phase consisted of 5 mM ammonium formate in water (A3) and acetonitrile (B3), with the same gradient program as above [27].

Mass spectrometric data were acquired using a Thermo Orbitrap Exploris 120 mass spectrometer (Thermo Fisher Scientific, USA) equipped with an electrospray ionization source, operating in both positive and negative ion modes. Spray voltages were set at 3.50 kV (positive) and − 2.50 kV (negative). Sheath and auxiliary gas flows were 40 arb and 10 arb, respectively, with a capillary temperature of 325 °C. Full MS1 scans were performed at a resolution of 60,000 over an m/z range of 100–1000. Higher-energy collisional dissociation (HCD) was used for MS2 fragmentation with a collision energy of 30%, acquiring data at a resolution of 15,000. The top 4 most intense ions were selected for fragmentation, while unwanted MS/MS events were excluded via dynamic exclusion [28].

### Statistical analysis

Data were analyzed using one-way analysis of variance (ANOVA) followed by Dunnett’s, Tukey’s, or Sidak’s multiple comparison tests. Results are presented as mean ± standard deviation (x̄ ± s). All statistical analyses were performed using GraphPad Prism software, version 8.0 (La Jolla, CA, USA). A p-value of less than 0.05 was considered statistically significant.

## Results

### GQBZP improves 5-FU induced IM in mice

**Fig. 2:**
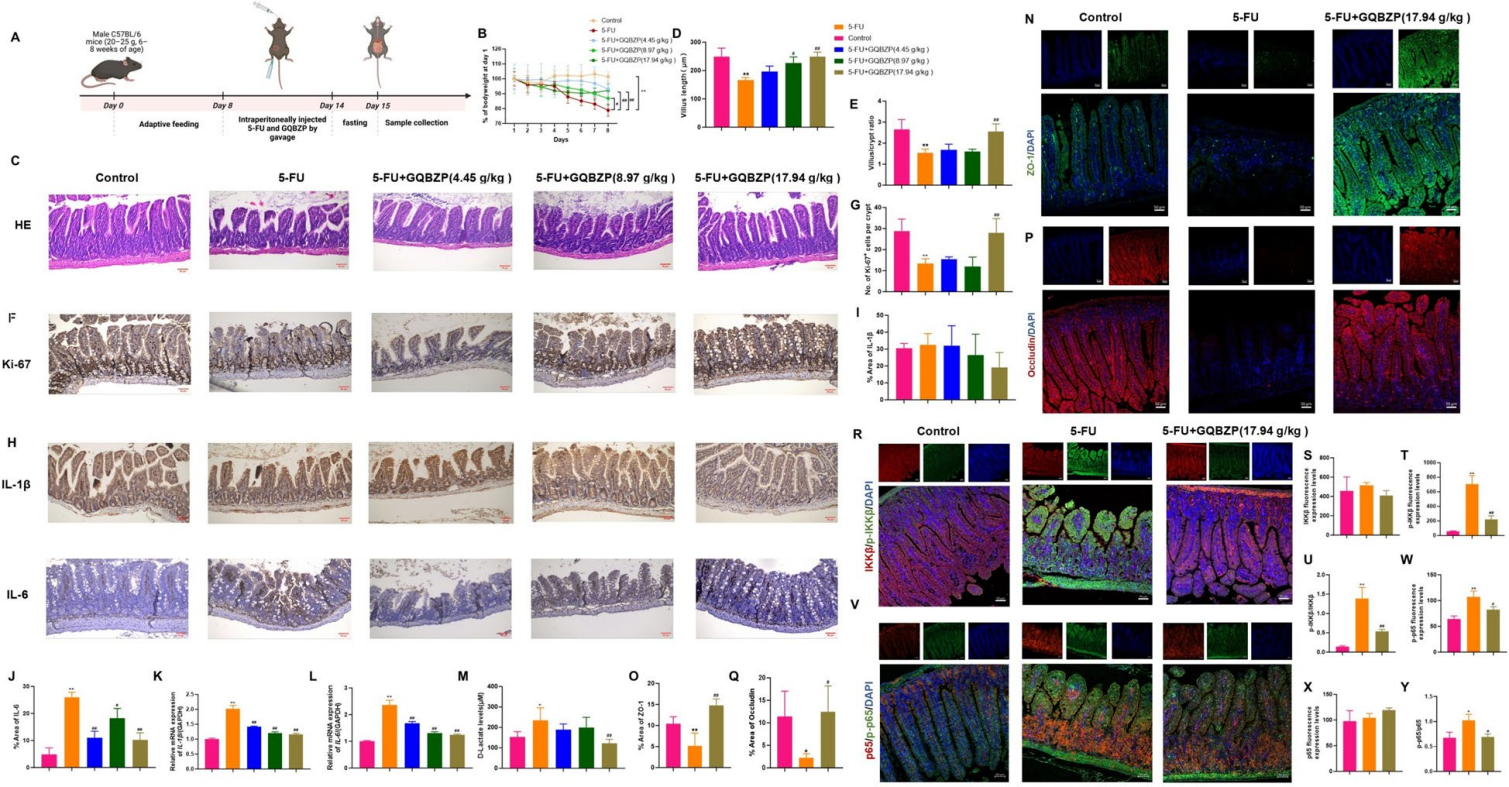
GQBZP can alleviate intestinal injury induced by 5-FU in mice. **A** Schematic diagram of experimental procedure. **B** Percentage changes in body weight of mice in each group (n = 7, per group). **C** Jejunal tissue was observed by H&E staining (scale = 50 μm). **D** Quantitative analysis of villus ratio in jejunal crypt (n = 3, per group). **E** Statistics of villus length in jejunal crypts (n = 3, per group). **F–G** Immunohistochemistry of Ki-67 in jejunal tissue and their statistics (n = 3, per group, scale = 50 μm). **H–J** Immunohistochemistry of IL-1β and IL-6 in jejunal tissue and their statistics (n = 3, per group, scale = 50 μm). **K–L** The mRNA expression levels of *IL-6* and *IL-1β* were quantified by RT-qPCR (n = 3, per group). **M** Serum D-lactic acid content of mice in each group (n = 5, per group). **N–Q** Average fluorescence intensity statistics of ZO-1 and Occludin (n = 5, per group, scale = 50 μm). **R–U** IKKβ and p-IKKβ immunofluorescence assay, and their statistics (n = 3, per group, scale = 50 μm). **V**–**Y** p65 and p-p65 immunofluorescence assay, and their statistics (n = 3, per group, scale = 50 μm). ^*^p < 0.05, ^**^p < 0.01, compared with control group; ^#^*p* < 0.05, ^##^*p* < 0.01, compared with the 5-FU group

C57BL/6 mice received intraperitoneal injections of 5-FU for 7 days while simultaneously being administered GQBZP via oral gavage (Fig. 2A). As shown in Fig. 2B, GQBZP at all three doses alleviated the weight loss caused by 5-FU, with the low-dose and high-dose groups exhibiting more pronounced protective effects. Compared to the control group, mice in the 5-FU group exhibited pathological changes in the jejunum, including reduced villus height and density, blunted villi, loss or necrosis of crypt structures, increased vacuolization, infiltration of inflammatory cells, and partial submucosal edema. Treatment with GQBZP significantly ameliorated these 5-FU-induced histopathological injuries in a dose-dependent manner, with the high-dose group showing the most complete recovery of villus architecture (Fig. 2C–E). Ki-67 immunohistochemistry of the jejunum revealed that 5-FU markedly suppressed intestinal crypt proliferation, whereas GQBZP partially restored this proliferative capacity, with the high-dose group displaying the most robust restoration (Fig. 2F–G). Compared to the control group, jejunal expression of IL-1β and IL-6 was significantly upregulated at both mRNA and protein levels following 5-FU exposure; in contrast, GQBZP effectively attenuated this pro-inflammatory response, with the high-dose group exerting the strongest inhibitory effect (Fig. 2H–L). Serum D-lactate levels were elevated by 5-FU treatment, and this increase was significantly reduced by GQBZP administration, with the high-dose group exhibiting the most pronounced effect (Fig. 2M). Furthermore, immunofluorescence analysis demonstrated disruption of tight junction integrity in the jejunum after 5-FU treatment, evidenced by discontinuous ZO-1 and Occludin expression patterns. In contrast, high-dose GQBZP restored the continuous, linear localization of these barrier proteins along the epithelial border (Fig. 2N–Q). Collectively, these findings demonstrate that high-dose GQBZP confers the greatest protection against 5-FU-induced intestinal mucositis in mice. Accordingly, the high-dose regimen was employed for follow-up mechanistic investigations.

Given the close association between the IKKβ/NF-κB signaling pathway and inflammatory responses, we conducted immunofluorescence experiments to assess the expression of this pathway in mouse jejunal tissues. The results showed that, compared to the control group, 5-FU treatment significantly increased the fluorescence intensity of p-IKKβ and p-p65, along with elevated p-IKKβ/IKKβ and p-p65/p65 ratios. Treatment with GQBZP effectively attenuated these changes (Fig. 2R–Y). These findings suggest that GQBZP alleviates 5-FU-induced inflammation and intestinal barrier damage in jejunal tissues, potentially through modulation of the IKKβ/NF-κB signaling pathway.

### GQBZP suppressed M1 polarization in the jejunum of mice with 5-FU-induced IM

**Fig. 3:**
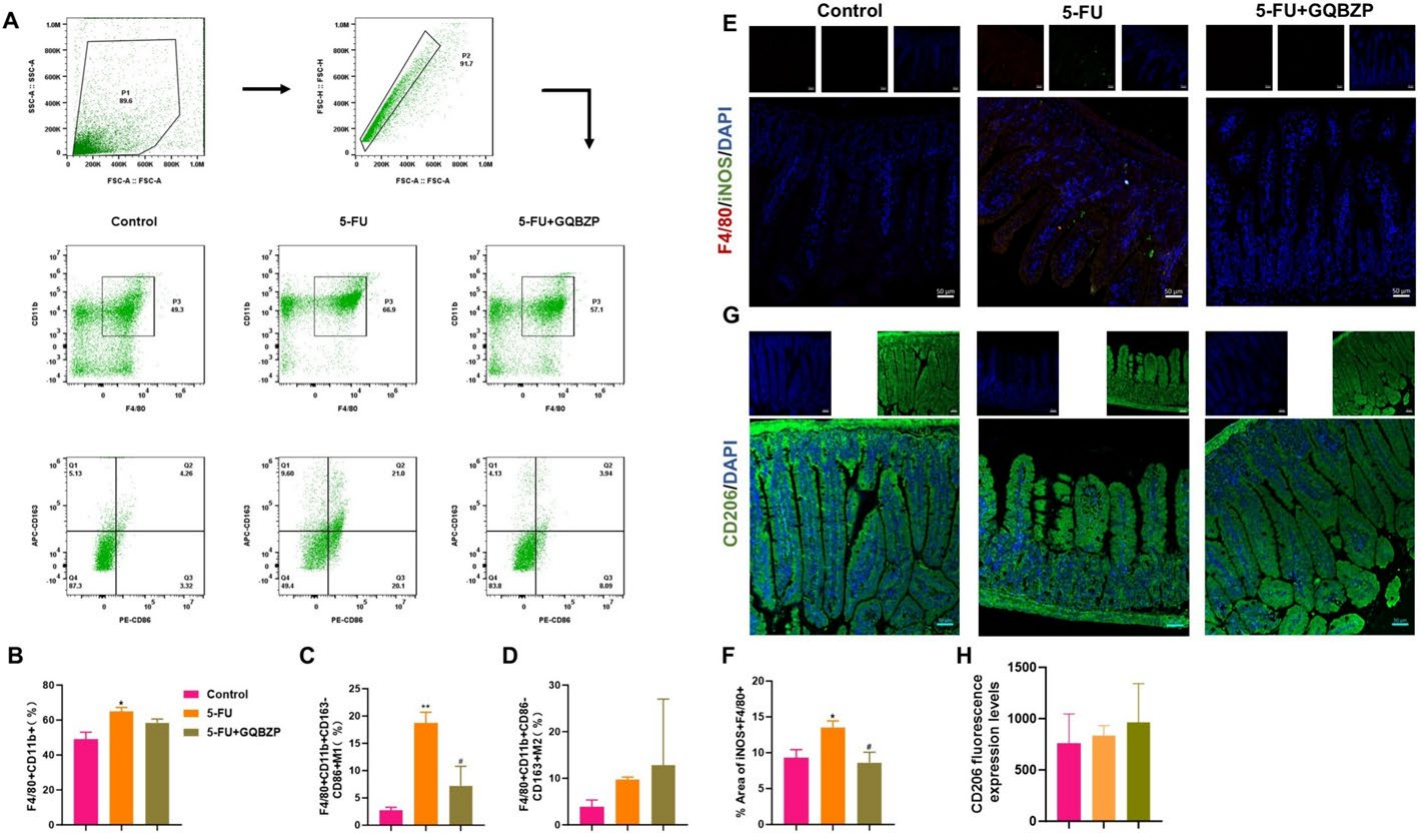
GQBZP inhibits 5-FU-induced jejunal M1 polarization in IM mice. **A–D** F4/80 and CD11b labeled intestinal macrophages, CD86 labeled M1, CD163 labeled M2 by flow cytometry, and their statistics (n = 3, per group). **E, F** F4/80 and iNOS labeled M1 by immunofluorescence, and their statistics, (n = 3, per group). **G, H** CD206 labeled M2 by immunofluorescence, and their statistics (n = 6, per group). One-way ANOVA with Dunnett’s multiple comparisons test. ^*^p < 0.05, ^**^p < 0.01, compared with control group; ^#^*p* < 0.05, ^##^*p* < 0.01, compared with the 5-FU group

It has been demonstrated that the onset of inflammation in CIM is associated with the process of macrophage polarization within the intestinal lamina propria [29]. Furthermore, inhibition of IKKβ/NF-κB activation has been shown to reverse M1 macrophage polarization and alleviate inflammatory responses [8]. We analyzed M1/M2 macrophage populations in mouse jejunal tissues undergoing 5-FU treatment by flow cytometry. Compared to the control group, 5-FU-treated mice exhibited a significant increase in the proportion of CD11b⁺F4/80⁺ macrophages and CD11b⁺F4/80⁺CD86⁺ (M1-like) cells. Although GQBZP treatment reduced the proportions of CD11b⁺ and F4/80⁺ cells relative to the 5-FU group, these changes were not statistically significant. Importantly, GQBZP significantly decreased the 5-FU-induced increase in CD11b⁺F4/80⁺CD86⁺ cells (Fig. 3A–C). No significant differences in CD163⁺ (M2 marker) expression were observed among the groups (Fig. 3D). Immunofluorescence analysis of jejunal tissues confirmed that 5-FU markedly increased the co-expression of F4/80 and iNOS (an M1 marker) compared to controls, and this effect was partially reversed by GQBZP treatment (Fig. 3E, F). Meanwhile, expression of CD206 (an M2 marker) remained unchanged across all groups (Fig. 3G, H). These findings suggest that the anti-inflammatory effects of GQBZP in 5-FU-induced IM are linked to the inhibition of M1 macrophage polarization in jejunal tissue, while neither 5-FU nor GQBZP significantly influences M2 polarization of intestinal macrophages.

### Virtual screening of potential IKKβ-targeting active compounds from GQBZP

Targeting IKKβ is an effective strategy for inhibiting the NF-κB signaling pathway and M1 macrophage polarization. To identify potential active compounds in GQBZP that target IKKβ, we performed molecular docking of 1051 GQBZP compounds with the IKKβ protein structure. Initial screening was conducted using Lipinski's Rule of Five criteria (molecular weight < 500, H-bond donors < 5, H-bond acceptors < 10, logP < 5, and rotatable bonds ≤ 10). Based on the monarch-minister-assistant-guide principle within GQBZP and purchasability, seven compounds with the highest docking scores were selected for further experimental validation (Table 1).

**Table 1:** IKKβ docking profiles and physicochemical characterization of GQBZP’s six lead compounds

Compound	Docking score	MW	HBA	HBD	logPo/w	Rotor	Latin name of the herb
Rhamnocitrin	− 8.21	316.27	5.25	3.00	1.20	5.00	Astragalus membranaceus (Fisch.) Bunge
Naringenin	− 8.18	272.26	4.00	2.00	1.66	3.00	*Citrus reticulata* Blanco
Carvacrol	− 7.77	150.22	0.75	1.00	3.29	2.00	*Citrus reticulata* Blanco
Toralactone	− 8.50497	272.26	3.75	3.00	2.155	1.00	Rheum palmatum L
Scopoletol	− 7.70	192.17	4	1	0.84	2	Atractylodis macrocephalae rhizoma
Akridin	− 7.60	179.22	1.00	0.00	3.40	0	Atractylodis macrocephalae rhizoma
Liquiritigenin	− 8.90	256.26	4.25	2.00	1.72	2.00	Glycyrrhiza uralensis Fisch. ex DC

HBA, hydrogen bond acceptors; HBD, hydrogen bond donors; MW, molecular weight; logPo/w, lipid-water partition coefficient; Rotor, number of rotatable bonds

### Compounds in GQBZP targeting IKKβ ameliorate 5-FU-Induced HIECs injury

**Fig. 4:**
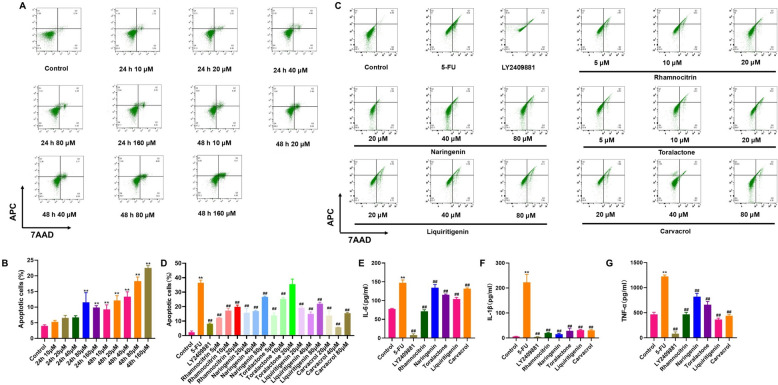
Active compounds targeting IKKβ in GQBZP can alleviate 5-FU-induced HIECs injury. **A, B** Flow cytometry was used to detect the apoptosis of HIECs and its statistical significance after 24 and 48 h of intervention with different concentrations of 5-FU (n = 3, per group). **C, D** Flow cytometry was used to detect the apoptosis and statistical significance of HIECs induced by 5-FU after 48 h of intervention with active compounds targeting IKKβ in GQBZP and LY2409881 (n = 3, per group). **E–G** The levels of inflammatory factors IL-6, IL-1β and TNF-α in the culture medium supernatant of each group were measured by ELISA (n = 5, per group). One-way ANOVA with Dunnett’s multiple comparisons test. ^*^p < 0.05, ^**^p < 0.01, compared with control group; ^#^*p* < 0.05, ^*##*^*p* < 0.01, compared with the 5-FU group

Firstly, we established a model of 5-FU-induced injury in HIECs. The CCK-8 assay demonstrated that 5-FU significantly inhibited HIEC proliferation in a time- and dose-dependent manner (Fig. S1A–C). Apoptosis analysis revealed that treatment with 80 μM and 160 μM 5-FU induced apoptosis rates exceeding 15% after 48 h (Fig. 4A–B). Based on these results, an incubation with 80 μM 5-FU for 48 h was selected to establish the HIEC injury model.

During CCK-8 screening, Akridin and Scopoletin were excluded due to their significant inhibitory effects on HIEC proliferation (Fig. S2). In contrast, the IKKβ inhibitor LY2409881 and five GQBZP-derived compounds targeting IKKβ were identified as protective agents for HIECs. Based on cell viability assays, the following concentrations were used in subsequent experiments: LY2409881 (10 µM); Rhamnocitrin and Toralactone (5, 10, and 20 µM); and Naringenin, Liquiritigenin, and Carvacrol (20, 40, and 80 µM).

Compared to the control group, 5-FU treatment significantly increased apoptosis in HIECs and elevated IL-6, IL-1β, and TNF-α levels in the culture media. Treatment with LY2409881 (10 µM), Rhamnocitrin (5–20 µM), Toralactone (5–10 µM), and Naringenin, Liquiritigenin, and Carvacrol (20–80 µM) significantly reduced apoptosis rates compared to 5-FU alone (Fig. 4C–D). Furthermore, HIECs treated with the optimal concentrations determined from apoptosis assays, LY2409881 (10 µM), Rhamnocitrin (10 µM), Toralactone (10 µM), Naringenin (40 µM), Liquiritigenin (40 µM), and Carvacrol (40 µM), showed significantly decreased IL-6, IL-1β, and TNF-α levels compared to the 5-FU group (Fig. 4E–G). These results indicate that the specified GQBZP-derived compounds effectively mitigate 5-FU-induced inflammation and apoptosis in HIECs.

### GQBZP-derived active compounds targeting IKKβ inhibit M1 polarization in LPS + IFN-γ induced THP-1 macrophages

**Fig. 5:**
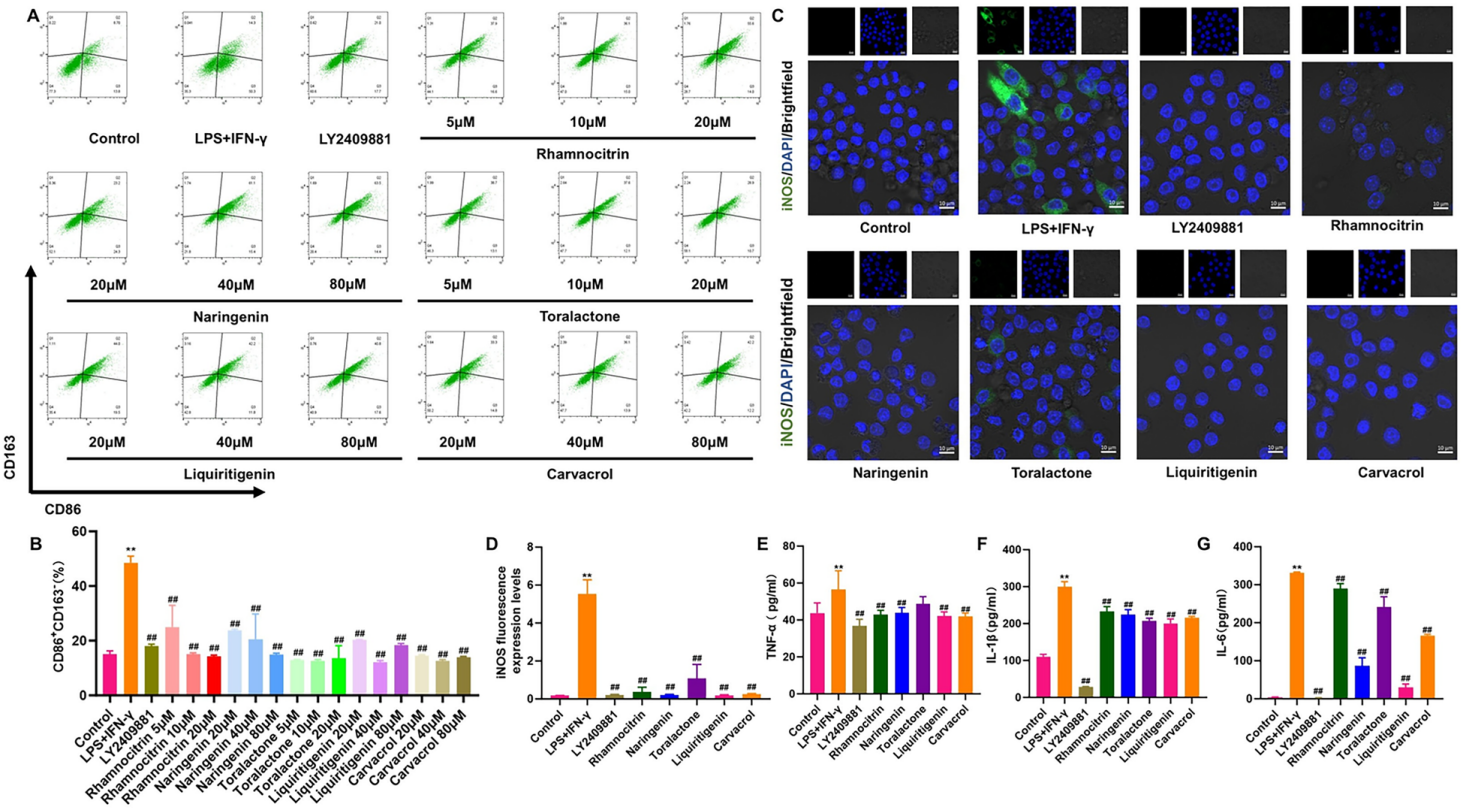
Active compounds targeting IKKβ in GQBZP reduce the M1 polarization induced by LPS + IFN-γ in THP-1 cells. **A, B** PE-CD86 labeled M1, APC-CD163 labeled M2, and their statistics (n = 3, per group). **C, D** iNOS labeled M1 by immunofluorescence, and their statistics (n = 3, per group). **E–G** The levels of IL-1β, TNF-α and IL-6 in each group as detected by ELISA (n = 5, per group). One-way ANOVA with Dunnett’s multiple comparisons test. ^*^p < 0.05, ^**^p < 0.01, compared with control group; ^#^*p* < 0.05, ^##^*p* < 0.01, compared with the LPS + IFN-γ group

Following induction with LPS and IFN-γ, THP-1 cells were treated with a concentration gradient of the previously identified compounds. Flow cytometry analysis revealed a significant increase in the proportion of CD86⁺ CD163^−^ cells in the LPS + IFN-γ group compared to the control group, indicating M1 macrophage polarization. Treatment with 10 μM LY2409881, Rhamnocitrin (5–20 μM), Toralactone (5–20 μM), and Naringenin, Liquiritigenin, or Carvacrol (each at 20–80 μM) significantly reduced the proportion of CD86⁺CD163^−^ cells compared to the LPS + IFN-γ group (Fig. 5A–B). Similar results were observed by immunofluorescence analysis of the M1 macrophage marker iNOS (Fig. 5C–D). The levels of inflammatory cytokines IL-1β, TNF-α, and IL-6 in the cell culture supernatants were significantly elevated in the LPS + IFN-γ group compared to controls. Treatment with 10 μM LY2409881, 10 μM Rhamnocitrin, 10 μM Toralactone, 40 μM Naringenin, 40 μM Liquiritigenin, or 40 μM Carvacrol significantly decreased IL-1β, IL-6, and TNF-α levels relative to the LPS + IFN-γ group, although Toralactone had no significant effect on TNF-α (Fig. 5E–G). These findings demonstrate that the GQBZP-derived compounds targeting IKKβ can effectively attenuate LPS + IFN-γ-induced M1 polarization in THP-1 cells.

### Effects of GQBZP-derived active compounds targeting IKKβ on NF-κB activation in HIEC and THP-1 cells

**Fig. 6:**
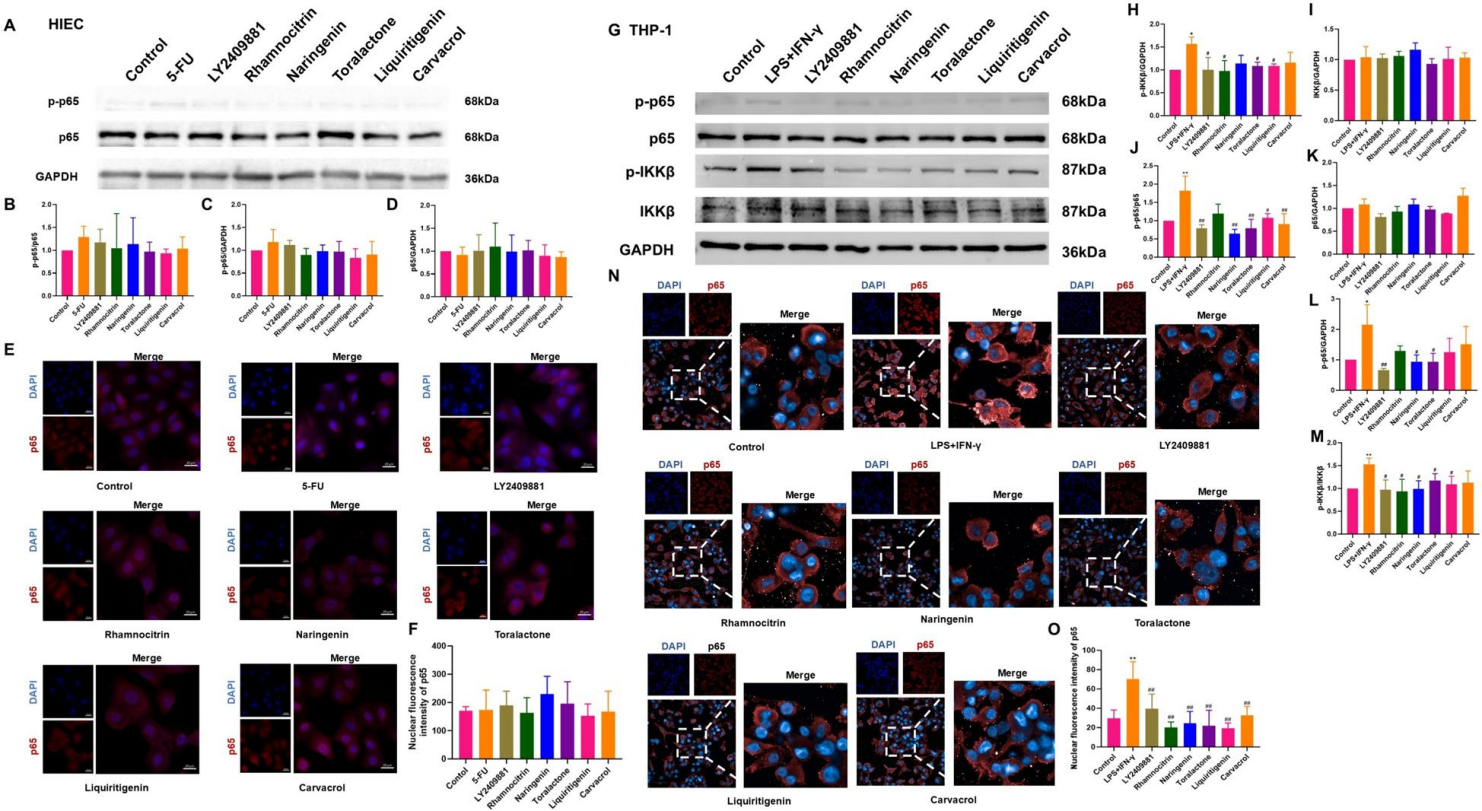
Active compounds targeting IKKβ in GQBZP inhibit the protein expression of IKKβ/NF-κB. **A–D** Detection of proteins p-p65 and p65 expression in HIECs by WB, and their statistics (n = 3, per group). **E, F** Immunofluorescence expression of p65 of HIECs, and their statistics (n = 3, per group). **G–M** Detection of proteins p-p65, p65, p-IKKβ, IKKβ expression in THP-1 cells by WB, and their statistics (n = 3, per group). **N, O** High Content Screening System fluorescence detection of expression of p65 in THP-1 cells, and their statistics (n = 3, per group). One-way ANOVA with Dunnett’s multiple comparisons test. ^*^p < 0.05, ^**^p < 0.01, compared with control group; ^#^*p* < 0.05, ^##^*p* < 0.01, compared with the 5-FU group or LPS + IFN-γ group

Initial assessment by Western blot and immunofluorescence showed that neither 5-FU nor the candidate compounds significantly affected p65 phosphorylation or nuclear translocation in the 5-FU-induced HIECs (Fig. 6A–F). In contrast, distinct effects were observed in THP-1 cells stimulated with LPS + IFN-γ. Compared to the control group, LPS + IFN-γ treatment significantly increased p-p65 and p-IKKβ protein levels, accompanied by elevated p-p65/p65 and p-IKKβ/IKKβ ratios (Fig. 6G–M). Treatment with LY2409881, Toralactone, and Naringenin significantly reduced p-p65 expression, while LY2409881, Toralactone, Naringenin, Liquiritigenin, and Carvacrol decreased the p-p65/p65 ratio compared to the LPS + IFN-γ group (Fig. 6G–J). Parallel inhibition of upstream signaling was evidenced by reduced p-IKKβ levels following treatment with LY2409881, Rhamnocitrin, Toralactone, and Liquiritigenin. Furthermore, LY2409881, Rhamnocitrin, Toralactone, Naringenin, and Liquiritigenin significantly decreased the p-IKKβ/IKKβ ratio (Fig. 6G, K–M). High-content screening supported these findings, showing a significant increase in p65 nuclear fluorescence intensity in LPS + IFN-γ-treated THP-1 cells compared to controls. Importantly, all tested compounds (LY2409881, Rhamnocitrin, Toralactone, Naringenin, Liquiritigenin, and Carvacrol) substantially attenuated the LPS + IFN-γ-induced nuclear accumulation of p65 (Fig. 6N–O). Together, these data demonstrate that specific GQBZP-derived active compounds effectively suppress the IKKβ/NF-κB signaling pathway in LPS + IFN-γ-treated THP-1 cells but fail to inhibit this pathway in 5-FU-induced HIECs.

### The targeting effects and interaction modes of Rhamnocitrin and Toralactone on IKKβ

**Fig. 7:**
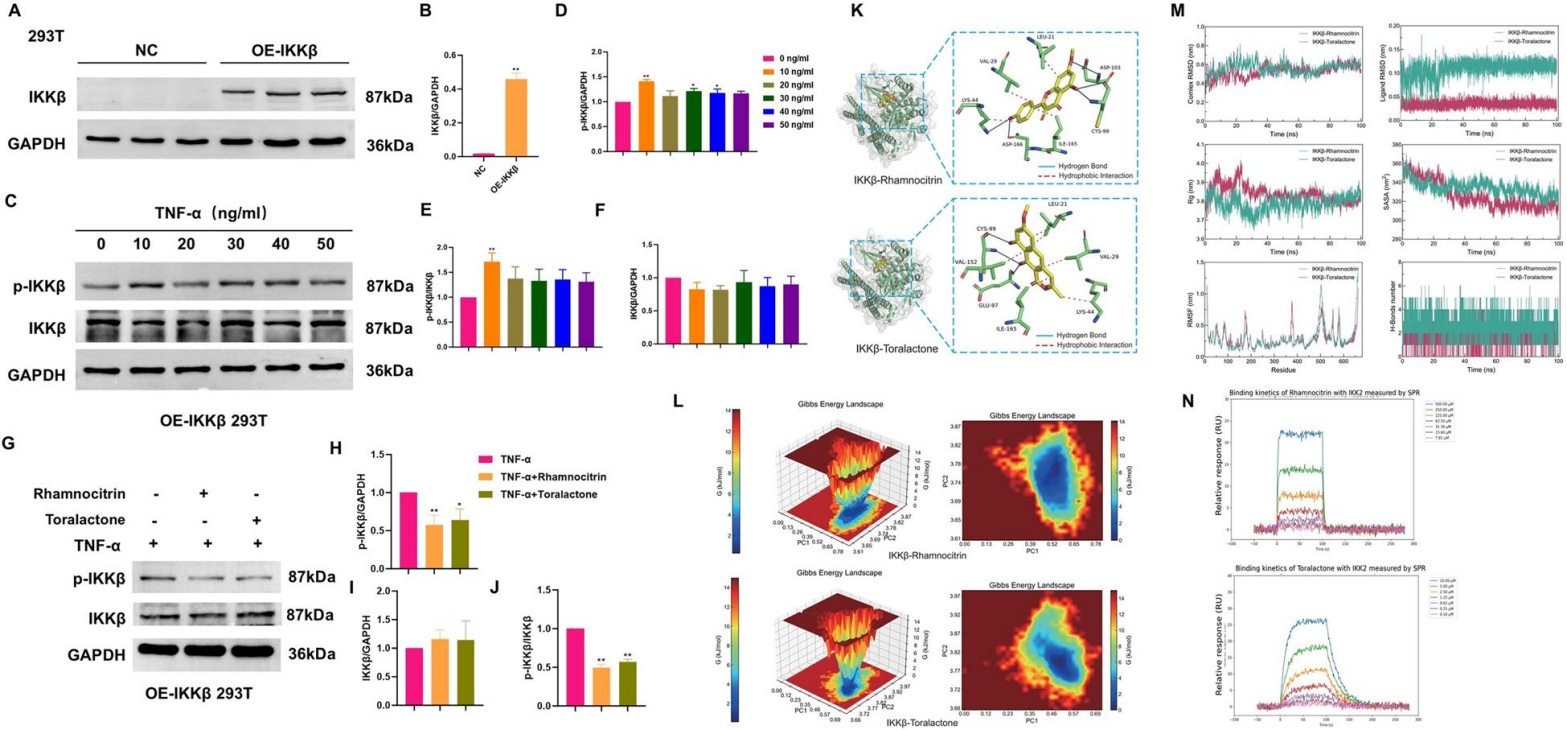
Validation of IKKβ Targeting by Rhamnocitrin and Toralactone. **A, B** WB was used to detect the expression of IKKβ in HEK293T cells transfected with the overexpression IKKβ (OE-IKKβ) plasmid, and their statistics (n = 3, per group). **C–F** WB was used to detect the expressions of p-IKKβ and IKKβ in OE-IKKβ HEK293T cells after 24 h of intervention with different concentrations of TNF-α, and their statistics (n = 3, per group). **G–J** The expression of p-IKKβ and IKKβ in OE-IKKβ HEK293T cells treated with Rhamnocitrin and Toralactone simultaneously with 10 ng/mL TNF-α was detected by WB, and their statistics (n = 3, per group). **K** Protein–Ligand Interaction Profiler (PLIP) analysis of Rhamnocitrin and Toralactone with IKKβ. **L** MD simulation analysis of IKKβ and Rhamnocitrin/Toralactone complex at 100 ns. **M **Gibbs free energy analysis of IKKβ-Rhamnocitrin/Toralactone complexes. **N** Binding kinetics of Rhamnocitrin and Toralactone with IKKβ by SPR. One-way ANOVA with Dunnett’s multiple comparisons test. ^*^p < 0.05, ^**^p < 0.01, compared with NC group, TNF-α 0 ng/mL group or TNF-α 10 ng/mL group

To further investigate the targeted regulation of IKKβ by these active compounds, an integrated analysis combining experimental data and literature review was performed based on previous studies. This analysis revealed that Rhamnocitrin and Toralactone remain understudied in the context of intestinal diseases, particularly regarding their interaction with IKKβ, warranting further investigation. The critical role of the IKKβ/NF-κB signaling pathway in macrophage M1 polarization is well established [30, 31]. However, due to the low transfection efficiency of THP-1 cells, HEK293T cells, known for their high transfection efficiency, were selected for validating receptor-ligand interactions in this study. Preliminary validation confirmed efficient transfection of the plasmid used for IKKβ overexpression (Fig. 7A–B). A dose–response assay with varying concentrations of TNF-α (0–50 ng/mL) showed that IKKβ phosphorylation was maximally induced at 10 ng/mL TNF-α (Fig. 7C–F). Subsequently, IKKβ-overexpressing HEK293T cells were treated with TNF-α (10 ng/mL) in combination with either Rhamnocitrin or Toralactone. Compared to TNF-α treatment alone, both the Rhamnocitrin + TNF-α and Toralactone + TNF-α groups exhibited a significant reduction in p-IKKβ expression, while total IKKβ protein levels remained unchanged. Correspondingly, the p-IKKβ/IKKβ ratio was also decreased (Fig. 7G–J).

PLIP analysis and MD simulations were employed to investigate the interactions and binding stability of Rhamnocitrin and Toralactone with IKKβ. Both compounds formed stable complexes with IKKβ through H-bonds and hydrophobic interactions (Fig. 7K). Comprehensive stability assessments, including RMSD, Rg, SASA, RMSF, hydrogen bonding patterns (Fig. 7M), Gibbs free energy (Fig. 7L), and binding free energy analyses (Table 2), demonstrated favorable conformational stability and biological activity throughout the simulations. To directly evaluate the effects of Rhamnocitrin and Toralactone on IKKβ, SPR assays were performed to detect molecular interactions. Based on the 1:1 Langmuir binding model, kinetic fitting of the SPR sensorgrams was performed, SPR quantified the binding affinities between IKKβ and the two compounds, revealing a high-affinity interaction for Toralactone (KD = 4.43e^−07^) and comparatively weaker binding for Rhamnocitrin (KD = 7.95e^−04^) (Fig. 7N and Table 3). These results suggest that Toralactone can directly target and inhibit IKKβ activation, whereas Rhamnocitrin may exert its regulatory effects via an indirect mechanism.

**Table 2:** MM/PBSA-calculated binding free energies of the complexes (kcal/mol)

Energy contributions	IKKβ-Rhamnocitrin	IKKβ-Toralactone
ΔVDWAALS	− 35.64	− 32.55
ΔEelec	− 19.87	− 11.37
ΔEGB	33.14	17.99
ΔEsurf	− 4.86	− 4.53
ΔGgas	− 55.51	− 43.92
ΔGsolvation	28.28	13.46
ΔTotal	− 27.23	− 30.46

ΔVDWAALS, van der Waals interaction energy; ΔEelec, electrostatic energy; ΔEGB, polar solvation energy (Generalized Born model); ΔEsurf, nonpolar solvation energy; ΔGgas, gas-phase free energy; ΔGsolvation, solvation free energy; ΔTotal, total binding free energy

**Table 3:** Binding kinetic affinity of IKKβ with Toralactone and Rhamnocitrin

Ligand	Ligand Conc	Level (RU)	Analyte	Analyte Conc	Ka (1/Ms)	Kd (1/s)	KD (M)	Chi^2^ (RU^2^)
IKK2	20 μg/mL	12,600	Toralactone	(0.16–10) μM	4.83e + 04	2.14e-02	4.43e-07	0.98
IKK2	20 μg/mL	12,600	Rhamnocitrin	(7–500) μM	4.93e + 02	3.92e-01	7.95e-04	0.98

ka(1/Ms), Association rate constant; kd(1/s), Dissociation rate constant; KD (M), Dissociation constant (a smaller KD indicates stronger binding affinity); Chi^2^(RU^2^), Goodness-of-fit value (values close to 1 indicate a high-quality fitting of the kinetic data)

## Discussion

5-FU, a widely used chemotherapeutic agent for solid tumors, inhibits tumor growth by disrupting DNA synthesis [32]. However, due to its lack of cellular selectivity, 5-FU also damages normal intestinal mucosa, causing inflammation, barrier dysfunction, and the onset of CIM. Currently, no specific therapeutic agents for CIM are available in clinical practice [33]. Although GQBZP has been shown to protect against intestinal injury caused by Ara-C and radiation, its efficacy against 5-FU-induced IM and its precise mechanisms, particularly its regulatory role in the intestinal immune microenvironment, remain largely unknown.

CIM is a complex pathological process involving all intestinal mucosal cells, primarily characterized by the amplification of inflammatory cascades [34]. Overactivation of the NF-κB pathway plays a central role in driving this progression [33], amplifying inflammatory signals that lead to dysregulation of the immune microenvironment. Key features include macrophage infiltration, skewed polarization, and elevated release of pro-inflammatory cytokines such as IL-6 and TNF-α. Our study demonstrated that 5-FU treatment activates the NF-κB pathway and significantly increases M1 macrophage accumulation in jejunal tissue, implicating inflammatory injury as a core mechanism. Notably, no increase in M2 macrophage polarization was observed, which may be explained by the anti-inflammatory and reparative functions of M2 macrophages occurring primarily during later stages of injury [35]. Since our observation points were concentrated in the acute inflammatory phase, this dynamic change may have been missed. Future studies should systematically track the temporal shifts in macrophage subpopulations to fully elucidate GQBZP’s role in the immune-epithelial repair network. Overall, these findings suggest that GQBZP alleviates 5-FU-induced IM primarily by inhibiting the IKKβ/NF-κB pathway and modulating macrophage polarization.

IKKβ, as an upstream kinase of NF-κB, promotes NF-κB nuclear translocation and subsequent transcription of inflammatory cytokines upon phosphorylation [28]. Increasing evidence highlights natural product-derived IKKβ inhibitors, such as Schisandrin A [36] (an allosteric inhibitor), brazilin-based nanomedicine (BX-Ce NPs) [37], and Glyasperin A [38], as promising therapeutic agents for precisely targeting NF-κB-driven pathologies. This underscores the translational potential of IKKβ as a therapeutic target in inflammation-related diseases. TCM, characterized by multi-component and multi-target properties, offers a rich source of such agents. In this study, molecular docking combined with HIEC proliferation assays identified five potential IKKβ-targeting bioactive compounds from GQBZP: Rhamnocitrin, Toralactone, Naringenin, Liquiritigenin, and Carvacrol. In LPS + IFN-γ-stimulated THP-1 macrophages, these compounds significantly attenuated CD86⁺ M1 polarization, reduced iNOS expression, and inhibited secretion of TNF-α, IL-1β, and IL-6. Simultaneously, they alleviated apoptosis and proinflammatory cytokine secretion in 5-FU-damaged HIECs, demonstrating preliminary evidence of dual activity in modulating macrophage inflammatory phenotypes and protecting intestinal epithelium. Notably, these two cell types exhibited distinct NF-κB pathway responses: LPS + IFN-γ activated the IKKβ/NF-κB pathway in THP-1 macrophages, which was effectively inhibited by the compounds. Conversely, 5-FU exposure did not induce phosphorylation of p65 or its nuclear translocation in HIECs, indicating a lack of significant NF-κB activation. This aligns with prior findings that NF-κB activation in ulcerative colitis patients’ gut is largely confined to lamina propria macrophages [39], with activation levels correlating positively with macrophage-derived inflammatory cytokine concentrations [40]. Mouse models further show that intestinal epithelial cells do not directly respond to LPS; instead, lamina propria macrophages detect LPS via TLR4, secrete TNF, and thereby indirectly initiate epithelial NF-κB signaling [41]. Coupled with the characteristically low expression of TLR family members (notably TLR4 and TLR5) in the small intestinal epithelium [42], this explains why 5-FU alone cannot directly trigger NF-κB activation in these cells due to insufficient upstream receptor recognition. Thus, the core protective mechanism of GQBZP appears twofold: first, it directly mitigates 5-FU-induced inflammatory injury and apoptosis in intestinal epithelial cells through pathways independent of IKKβ/NF-κB; second, it indirectly protects the epithelium by inhibiting macrophage M1 polarization and reducing inflammatory cytokine secretion via downregulation of the IKKβ/NF-κB pathway. It is important to note that the in vitro macrophage model employed classical LPS/IFN-γ stimulation, which reliably reflects IKKβ/NF-κB-regulated inflammatory responses. However, this approach does not fully recapitulate the complexity of the intestinal inflammation induced by chemotherapy drugs such as 5-FU in the tumor microenvironment [43, 44], lacking specific molecular patterns such as damage-associated molecular patterns found in the 5-FU-damaged microenvironment. Future studies using conditioned media from 5-FU-injured epithelium or specific alarm stimuli will enable the development of models more representative of the actual disease context.

Previous studies have demonstrated that compounds such as Naringenin [45, 46], Liquiritigenin [47], and Carvacrol [48] exert beneficial effects in intestinal diseases primarily by modulating NF-κB-related pathways. However, mechanistic insights and translational potential regarding Rhamnocitrin and Toralactone in intestinal pathologies remain limited. Accordingly, this study focused on these two compounds. Using a multifaceted approach, including PLIP analysis, MD simulations, Gibbs free energy calculations, and experiments with IKKβ-overexpressing cells, we confirmed that both compounds can modulate IKKβ phosphorylation levels. Notably, SPR analysis revealed a striking difference in their binding affinities to IKKβ, with an approximately 1800-fold disparity. The K_D is a critical parameter for assessing ligand-target binding stability. Toralactone exhibited a sub-micromolar K_D (~ 443 nM), indicative of stable complex formation with IKKβ at physiologically relevant concentrations, consistent with characteristics of direct inhibitors [49]. Conversely, Rhamnocitrin’s K_D approached the millimolar range (~ 795 μM), far exceeding concentrations achievable in vivo. According to established standards for biomolecular interactions, this suggests that Rhamnocitrin is unlikely to act via direct binding to IKKβ. Interestingly, Rhamnocitrin significantly inhibited p-IKKβ levels at concentrations well below its K_D. This aligns with prior literature indicating that functional inhibitory effects do not always correlate with direct binding affinity [50]. This observation suggests that Rhamnocitrin may exert its effects indirectly by modulating upstream signaling pathways or via off-target mechanisms rather than through direct interaction with IKKβ. These findings illustrate the complexity of the multi-component, multi-target mechanisms characteristic of TCM formulations. While this study preliminarily identifies Toralactone as a direct IKKβ inhibitor, further investigation is required to elucidate the roles of other active components, potential synergistic interactions, and in vivo pharmacokinetic properties. Future research integrating multidimensional approaches such as network pharmacology and metabolomics may provide deeper insights into the material basis of GQBZP’s efficacy and the synergistic mechanisms among its constituents.

## Conclusion

**Fig. 8:**
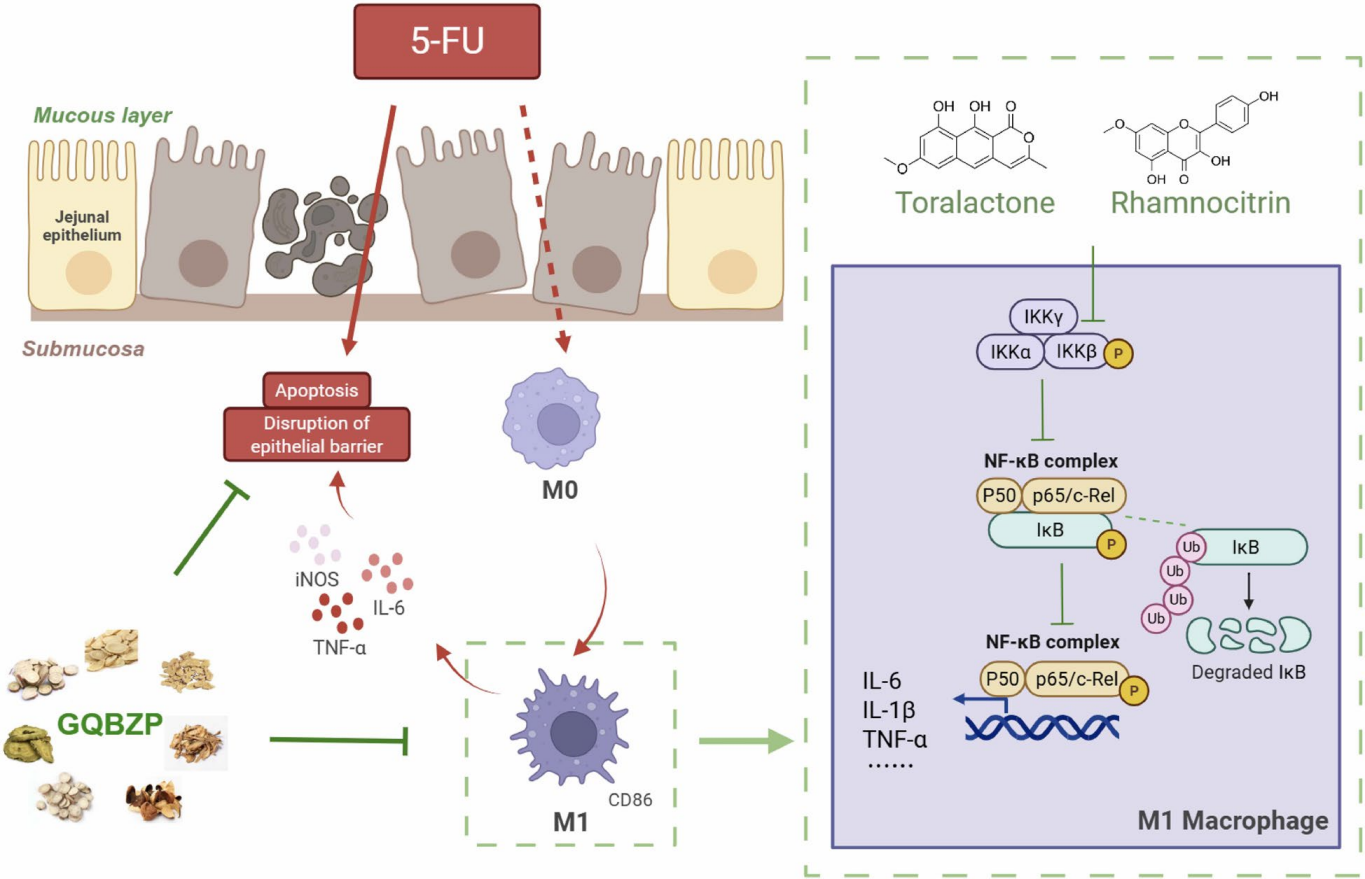
Mechanism of GQBZP in the treatment of 5-FU-induced IM. Red solid lines represent the side effects of 5-FU on the intestine, manifested as macrophage M1 polarization, IKKβ activation, and intestinal barrier impairment. The activation of IKKβ increases the number of M1 macrophages and accelerates excessive inflammatory responses, both of which lead to intestinal mucositis. Green solid lines indicate that GQBZP can alleviate inflammatory responses by inhibiting the activation of the IKKβ signaling pathway and remodeling macrophage polarization, as well as protect intestinal epithelial cells in an IKKβ/NF-κB pathway-independent manner, ultimately ameliorating 5-FU-induced IM

In summary, GQBZP alleviates 5-FU-induced IM by dual mechanisms: it inhibits M1 polarization of intestinal mucosal macrophages through an IKKβ/NF-κB pathway-dependent mechanism, while concurrently protecting small intestinal epithelial cells via an IKKβ/NF-κB-independent pathway (Fig. 8). Among its active components, Toralactone acts as a direct IKKβ inhibitor, whereas Rhamnocitrin likely exerts its effects indirectly by modulating upstream signaling pathways or through off-target actions. These findings support the therapeutic potential of GQBZP for managing 5-FU-induced CIM.

## Supplementary Information

Supplementary Material 1

Supplementary Material 2

Supplementary Material 3

Supplementary Material 4

## Data Availability

No datasets were generated or analysed during the current study.

## Abbreviations

GQBZP: Guiqi Baizhu Prescription
5-FU: 5-fluorouracil
CIM: Chemotherapy-induced intestinal mucositis
IM: Intestinal mucositis
IKKβ: Inhibitor of Nuclear Factor κB Kinase β
HIEC: Human intestinal epithelial cell
LPS: Lipopolysaccharide
IFN-γ: Interferon-γ
MD: Molecular dynamics
SPR: Surface plasmon resonance
NF-κB: Nuclear Factor Kappa B
DNA: Deoxyribonucleic Acid
IL-1β: Interleukin-1β
IL-6: Interleukin-6
TNF-α: Tumor Necrosis Factor-α
iNOS: Inducible Nitric Oxide Synthase
TLR: Toll-Like Receptor
Ara-C: Cytarabine
GAPDH: Glyceraldehyde-3-phosphate dehydrogenase
p-IKKβ: phosphorylated Inhibitor of Nuclear Factor κB Kinase β
ZO-1: Zonula Occludens-1
UPLC-MS/MS: Ultra Performance Liquid Chromatography-Tandem Mass Spectrometry
HEK293T: Human embryonic kidney 293T
THP-1 cell: Human monocytic leukemia cell
PMA: Phorbol 12-Myristate 13-Acetate
DMSO: Dimethyl Sulfoxide
HCD: Higher-energy collisional dissociation
PLIP analysis: Protein-Ligand Interaction Profiler analysis
RMSD: Root Mean Square Deviation
Rg: Radius of Gyration
SASA: Solvent Accessible Surface Area
RMSF: Root Mean Square Fluctuation
TCM: Traditional Chinese Medicine
TLR4: Toll-Like Receptor 4
TLR5: Toll-Like Receptor 5
