# Supplementary material for: Guiqi Baizhu prescription attenuates 5-FU-induced intestinal mucositis by targeting IKKβ to inhibit M1 macrophage polarization
Source: Chin Med. 2026 Jul 16;21:194. doi: 10.1186/s13020-026-01406-z (PMC13374300; doi:10.1186/s13020-026-01406-z)
Supplement: Supplementary file 1 — Supplementary Material 1 [file 13020_2026_1406_MOESM1_ESM.pdf]

甘肃中医药大学动物实验伦理审查表

Animal Experimental Ethical Inspection
of Gansu University Of Chinese Medicine

编号	2022-159

申请人填写的相关信息 (Related information filled by applicant)					
	申请单位 Name of organization	甘肃中医药大学			
	申请人 Applicant	李亚玲			
	联系电话 Telephone	13919469095			
	申请日期 Applicantion date	2022 年 03 月 15 日			
	实验名称 Experiment title	基于 Keap1-Nrf2 和 NF-κB 通路探究健脾化瘀法增强抗氧化能力并抑制炎症反应治疗化疗肠黏膜炎的分子机制			
	拟实验时间 Experiment date	2024 年 07 月 01 日至 2026 年 09 月 30 日			
	拟实验场地 Experiment site	甘肃中医药大学实验动物中心			
使用动物情况	动物来源 Source of animal	甘肃中医药大学实验动物中心			
	品种品系 Species of strain	SD 大鼠<br>C57BL/6 小鼠	等级 Grade	SPF	
	数量 Number	♂ 100 只、♀ 0 只，大鼠 42 只，小鼠 58 只，共 100 只	规格（体重或年龄） Specifications	大鼠：200±10g<br>小鼠：18g-22g	

实验要点：包括研究方法概述、主要观测指标、实验结束后处死动物的方法等(Outline of experiments, experimental methods, observational index, executing animal method et. al)：

研究方法及主要观察指标：

（1）SPF 级 SD 大鼠用于归芪白术方含药血清的制备：标准条件下饲养和驯化一周，实验前禁食不禁饮 12h，将归芪白术方水提物按照体表面积换算后按 6.21g/kg 灌胃，分别于给药后 0、0.25、0.5、1.0、2.0、4.0、8.0h 经大鼠眼丛静脉采血 1mL，每个时间点采血 6 只大鼠，分离血清后保存在-80°C 中备用。主要检测血清中带测定化合物的含量。

（2）SPF 级 C57BL/6 小鼠用于体内实验评价归芪白术方小分子及配伍的活性：对实验小鼠进行皮下注射胃癌细胞，制备小鼠成瘤模型，对荷瘤成功的小鼠进一步建立 5-FU 致肠黏膜炎模型，具体为实验第 5-8d 连续 5 天腹腔注射浓度为 50mg/kg 的 5-FU，小分子干预组需于实验第 1-10d 分别灌胃小分子，期间观察并记录小鼠饮食、饮水、精神活动度、体重、腹泻次数等一般状况。于第 11d 处死小鼠，对瘤体测量大小、称重、制作切片用于 HE 染色，留取血和小肠组织用于后续检测。

主要观测指标：

制作切片形态检测和激光共聚焦检测蛋白表达，提取蛋白和 RNA，采用 WB、ELISA、流式细胞、qPCR 等检测 Keap1-Nrf2 和 NF-κB 通路及下游相关因子的表达。

实验动物的处死及尸体的处理：

3%戊巴比妥（30mg/Kg）腹腔内注射麻醉实验动物，颈椎脱臼处死，尸体交实验动物中心由甘肃省危险废弃物处置中心统一无害化处理。

申请者声明 Announcement of applicant	我将自觉遵守实验动物福利伦理原则，随时接受实验动物伦理委员会的监督与检查，如违反规定，自愿接受处罚。<br>（I will abide by the rules of animal experimental ethics, accept the supervision and inspection of the animal experimental ethics committee, and accept the punishment if any infringement.）<br><br>申请者签名：李亚玲<br>2022 年 3 月 16 日		
审查依据 Inspection contents	1. 该项目是否必须用实验动物进行实验，即能否用计算机模拟、细胞培养等方法替代动物或用低等动物替代高等动物进行实验(Does laboratory animal must be used in the project? Could other methods such as computer simulation. cell culture or using the low-grade animal instead of the high－grade animal?)<br>2. 表中所填申请人资格和所用动物的品种品系、质量等级、规格是否合适，能否通过改良设计方案或用高质量的动物来减少所用动物的数量(Are the qualification of applicant，species or strain，grade and specifications of animals suitable? Could the quantity of animals be reduced by improving the study design or using high quality animals?)<br>3. 能否通过改进实验方法、调整实验观测指标、改良处死动物的方法，来优化实验方案、善待动物(Could the study design and animal treatment be refined by ameliorating experimental method，adjusting observational index. executing animal method?)		
审查结果(是否同意申请人意见) Results of inspection	审查人意见 Attitude of Ethical Reviewer	符合 GB/T 35892-2018 实验动物 福利伦理审查指南<br><br>签名：张延英<br>2022 年 3 月 16 日	
	实验动物伦理委员会意见 Attitude of Animal Care Welfare Committee	同意审查人意见<br><br>甘肃中医药大学 实验动物伦理委员会<br>（盖章）<br>2022 年 3 月 16 日	
备注：Remark			

说明：
1. 编号由实验动物中心分配并填写。
2. 表格所有填写内容请用签字笔填写或电脑打印(签名处除外)。
3. 需随本表递交相关审查资料如实验方案、课题标书等。要求写明项目的意义、必要性、项目中有关实验动物的用途、饲养管理或实验处置方法、预期出现的对动物的伤害、处死动物的方法、项目进行中涉及动物福利和伦理问题的详细描述。
4. 本表一式两份，申请人一份，实验动物中心一份。
