# Supplementary figures and images for: Guiqi Baizhu prescription attenuates 5-FU-induced intestinal mucositis by targeting IKKβ to inhibit M1 macrophage polarization
Source: Chin Med. 2026 Jul 16;21:194. doi: 10.1186/s13020-026-01406-z (PMC13374300; doi:10.1186/s13020-026-01406-z)

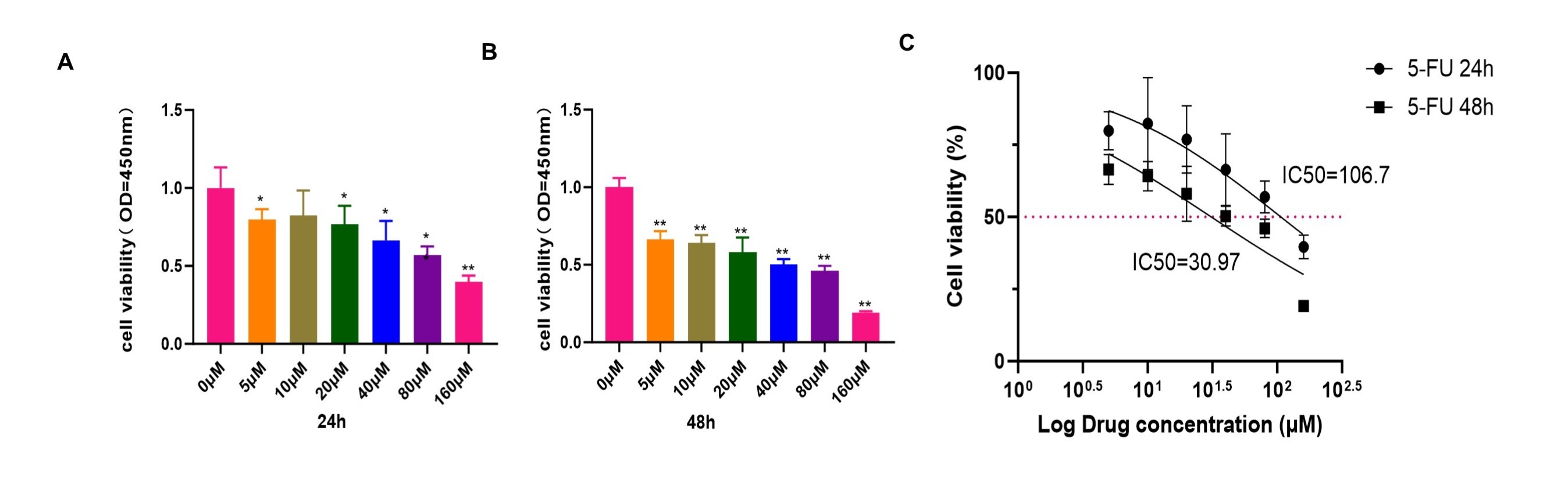

Supplement: Supplementary file 2 — Supplementary Material 2 [file 13020_2026_1406_MOESM2_ESM.jpg]

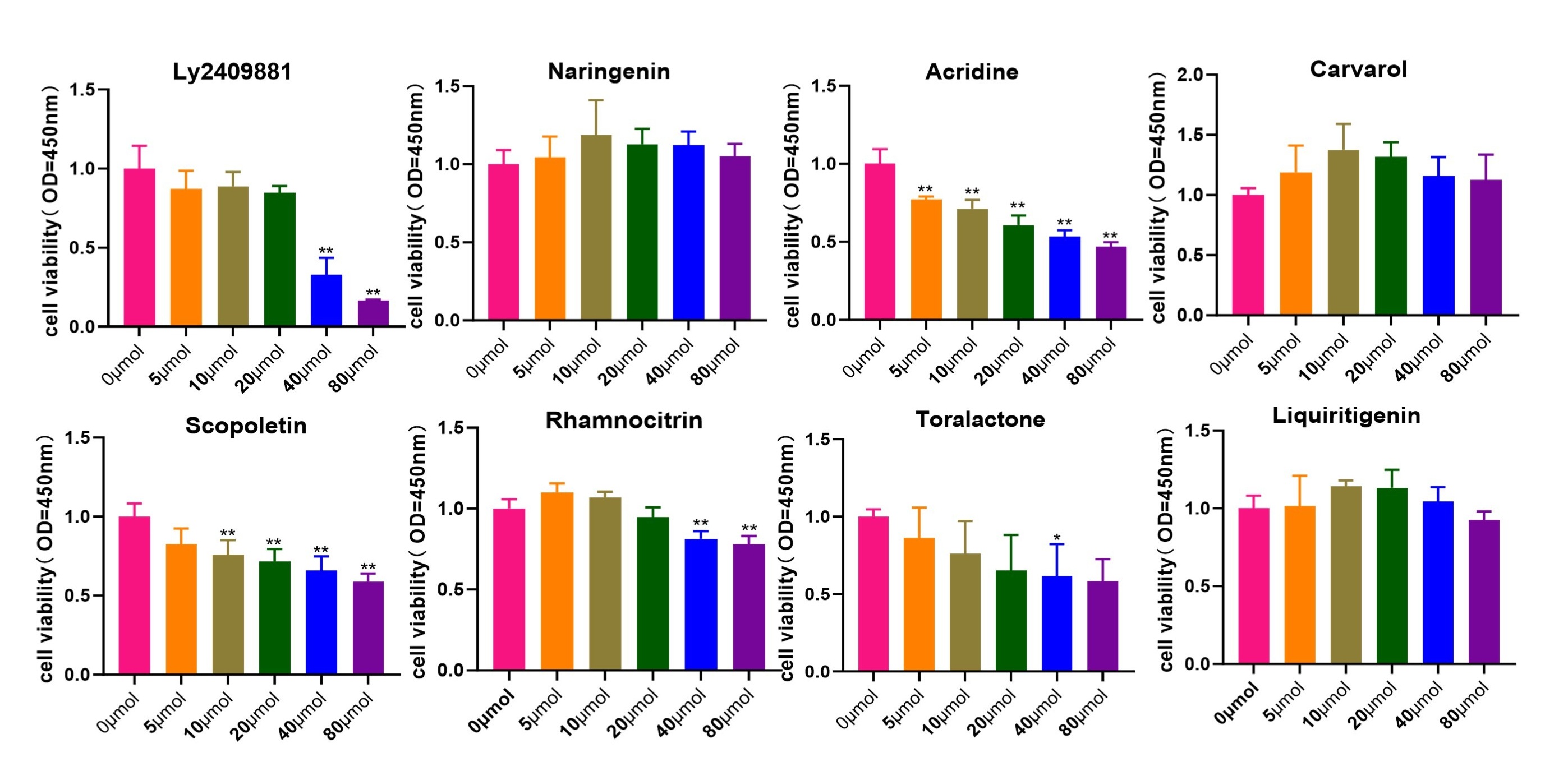

Supplement: Supplementary file 3 — Supplementary Material 3 [file 13020_2026_1406_MOESM3_ESM.jpg]
