# Supplementary material for: Guiqi Baizhu prescription attenuates 5-FU-induced intestinal mucositis by targeting IKKβ to inhibit M1 macrophage polarization
Source: Chin Med. 2026 Jul 16;21:194. doi: 10.1186/s13020-026-01406-z (PMC13374300; doi:10.1186/s13020-026-01406-z)
Supplement: Supplementary file 4 — Supplementary Material 4 [file 13020_2026_1406_MOESM4_ESM.docx]

Table S1: Identification of Representative Chemical Components in the Aqueous Decoction of GQBZP via UPLC-MS/MS

| t/min | Name | m/z | Exact  Mass | ppm | formula | Precursor  Type | Source (Herb) |
| --- | --- | --- | --- | --- | --- | --- | --- |
| 291.6 | Formononetin | 267.07 | 268.07 | 0.45 | C16H12O4 | [M-H]- | Astragali Radix et Rhizoma |
| 307.6 | Atractylenolide iii | 247.13 | 248.14 | 3.80 | C15H20O3 | [M-H]- | Atractylodis Macrocephalae Rhizoma |
| 424.4 | Emodin | 269.05 | 270.05 | 2.43 | C15H10O5 | [M-H]- | Rhei Radix et Rhizoma |
| 302.7 | Hesperidin | 655.19 | 610.19 | 4.37 | C28H34O15 | [M+HCOO]- | Citri Reticulatae Pericarpium |
| 294.6 | Naringin | 581.18 | 580.18 | 4.43 | C27H32O14 | [M+H]+ | Citri Reticulatae Pericarpium |
| 436.5 | Nobiletin | 403.14 | 402.13 | 6.05 | C21H22O8 | [M+H]+ | Citri Reticulatae Pericarpium |
| 273.5 | Paeoniflorin | 525.16 | 480.16 | 0.30 | C23H28O11 | [M+HCOO]- | Paeoniae Radix Alba |
| 308.1 | J17.727j | 514.19 | 496.16 | 2.13 | C23H28O12 | [M+NH4]+ | Paeoniae Radix Alba |
| 273.5 | Albiflorin | 479.15 | 480.16 | 3.92 | C23H28O11 | [M-H]- | Paeoniae Radix Alba |
| 482.7 | Ligustilide | 191.1072 | 190.242 | 1.004 | C12H14O2 | [M+H]+ | Angelicae Sinensis Radix |
| 395.8 | Liquiritigenin | 257.08 | 256.07 | 1.678 | C15H12O4 | [M+H]+ | Glycyrrhizae Radix et Rhizoma |
| 241.7 | Liquiritin | 419.13 | 418.15 | 8.95 | C21H22O9 | [M+H]+ | Glycyrrhizae Radix et Rhizoma |
| 297 | Glycyrrhizin | 821.40 | 822.40 | 0.74 | C42H62O16 | [M-H]- | Glycyrrhizae Radix et Rhizoma |

Table S2: Primer sequence

| Oligo Name | Sequence（5'to3'） |
| --- | --- |
| IL-6-Mice-F | CACTTCACAAGTCGGAGGCT |
| IL-6-Mice-R | CTGCAAGTGCATCATCGTTGT |
| IL-1β-Mice-F | GCAACTGTTCCTGAACTCAACT |
| IL-1β-Mice-R | ATCTTTTGGGGTCCGTCAACT |
| M-GAPDH-F | AGGTCGGTGTGAACGGATTTG |
| M-GAPDH-R | TGTAGACCATGTAGTTGAGGTCA |
